# Aminotransferases as causal factors for metabolic syndrome: A bidirectional Mendelian randomization study

**DOI:** 10.1371/journal.pone.0302209

**Published:** 2024-04-25

**Authors:** Meng Lingyu, Li Hongguang, Zhang Mingdong, Li Na, Liu Yahui

**Affiliations:** 1 Hepatobiliary and Pancreatic Surgery Department, General Surgery Center, First Hospital of Jilin University, Changchun, Jilin, China; 2 Office of Hospital Infection Control, The Third Affiliated Hospital of Changchun University of Chinese Medicine, Changchun, Jilin, China; 3 Faculty of Rehabilitation Medicine, Changchun University of Chinese Medicine, Changchun, Jilin, China; 4 Beihua University, Changchun, Jilin, China; Alexandria University, EGYPT

## Abstract

**Background:**

Circulating aminotransferases (ALT and AST) have been used as biomarkers for liver injury. The causal relationships between aminotransferases and metabolic syndrome remain ambiguous.

**Methods:**

We conducted bidirectional and multivariable Mendelian randomization (MR) analyses between aminotransferases and traits related to metabolic syndrome using genetic variants obtained from genome-wide association studies (GWASs). MR-PRESSO tests were adopted to remove outliers and eliminate pleiotropy. MR steiger tests were conducted to ensure the correct direction of the causal effects.

**Results:**

Both aminotransferases were risk factors for essential hypertension. ALT is a risk factor for type 2 diabetes. The bidirectional causal relationship between ALT and hyperglycemia, serum lipids, and obesity was demonstrated. The effect of fasting glucose on AST was demonstrated, while type 2 diabetes did not affect AST. The effect of HDL-C on ALT and the effect of triglycerides on AST were found in multivariable MR analyses.

**Conclusions:**

Our bidirectional MR analyses suggest that ALT and AST are causally associated with several metabolic syndrome-related traits, especially hypertension and type 2 diabetes. These findings highlight the potential role of aminotransferases as biomarkers and therapeutic targets for metabolic syndrome.

## Introduction

Metabolic syndrome was first described by Reaven in 1988 [1]. The most widely accepted definition of metabolic syndrome includes elevated waist circumference, elevated triglycerides, reduced HDL-C, elevated blood pressure and elevated fasting glucose [2]. If three out of five traits occurred, the diagnosis of metabolic syndrome was made. A rapid increase in metabolic syndrome has been observed in recent years. In China, between 1992 and 2002, the incidence of metabolic syndrome increased from 8 to 10.6% in urban areas and 4.9 to 5.3% in rural areas [3, 4]. Consequently, the early detection and prevention of metabolic syndrome is of vital importance.

Aminotransferases are enzymes that catalyze the transfer of amino groups between amino acids and α-keto acids. They are mainly expressed in the liver, where they participate in gluconeogenesis, ketogenesis, and amino acid metabolism. Aminotransferase levels are commonly used as biomarkers of liver injury and inflammation. However, an increasing number of people without liver disease could also have an elevated level of aminotransferases. An observational study suggests that fast-food-based hyperalimentation with a sedentary lifestyle would increase the circulating level of ALT without detectable injury to the liver [5]. Moreover, the relationship between ALT and metabolic syndrome was also observed in different cohorts [6–8]. However, the relationship between AST and metabolic syndrome remains uncertain. Furthermore, little effort has been made to investigate the association between aminotransferases and blood pressure. To address these issues, we carried out bidirectional Mendelian randomization analyses to test the causal relationships between aminotransferases (ALT, AST) and metabolic syndrome.

Mendelian randomization (MR) is a powerful method to test the causal effect of exposure on outcome using summary statistics of genome-wide association studies (GWAS). Compared to conventional randomized controlled trials, MR analysis is more suitable to investigate the long-term causal effect of risk factors on outcome due to the random assortment and lifelong effect of genetic variants. The single nucleotide polymorphisms (SNPs) extracted from different studies were used as instrumental variables in this study. Then, bidirectional MR analyses were conducted between aminotransferases and traits related to metabolic syndrome. These traits were divided into two categories: binary traits (essential hypertension, NAFLD, and type 2 diabetes) and continuous traits. The continuous traits were divided into hyperglycemia-related traits (fasting glucose, fasting insulin, two-hour post challenge glucose and HbA1c), obesity-related traits (waist-to-hip ratio and BMI) and serum lipids (HDL-C, LDL-C and triglycerides). Since the strong horizonal pleiotropy between the serum lipids cannot be easily eliminated, we carried out multivariable MR analysis to adjust the effect estimates of each lipid.

The purpose of this study was to describe and examine the causal relationship between aminotransferases and metabolic syndrome.

## Materials and methods

### Data source of GWAS summary statistics

To avoid overlapping samples between exposure and outcome, we extracted GWAS summary data from different consortia. We obtained GWAS summary data of ALT and AST from Neale lab analysis of UK biobank phenotypes, round 2 (http://www.nealelab.is/uk-biobank). A total of 470579 and 469030 individuals were included in the GWAS of ALT and AST, respectively. Quality control filters were applied to exclude SNPs with minor allele frequency < 0.01, Hardy-Weinberg equilibrium p-value < 1e-6, or imputation quality score < 0.8. Individuals included in the GWAS analysis of the UK biobank were restricted to European descent. The inverse rank normalized data were chosen for this MR study.

The GWAS summary data of essential hypertension and type 2 diabetes were extracted from FinnGen (http://r5.finngen.fi/). A total of 218754 individuals of Finnish ancestry, including 42857 cases and 162837 controls, were included in this GWAS of essential hypertension. The GWAS summary data of type 2 diabetes included 32469 cases and 183185 controls in total. Individuals with ambiguous gender high genotype missingness (5%), heterozygosity (+-4SD) and non-Finnish ancestry were excluded.

The GWAS summary data of hyperglycemia-related traits were obtained from the Meta-Analyses of Glucose and Insulin-related traits Consortium (MAGIC https://magicinvestigators.org/) [9, 10]. The GWAS analyses included 133010, 108557, 42854 and 123665 individuals for fasting glucose, fasting insulin two-hour postchallenge glucose and HbA1c, respectively [9, 10]. All individuals of this GWAS analysis were of European descent and were mostly adults. As few as 7872 and 7164 adolescents were included in the fasting glucose and fasting insulin analyses, respectively.

We obtained summary statistics of NAFLD from the research of Anstee [11]. They recruited 1483 cases from several leading European tertiary liver centers and 17781 controls from multiple sources. A total of 19264 individuals participated in the GWAS analysis. All participants were of European ancestry.

We extracted GWAS summary data of traits related to obesity, including BMI and waist-to-hip ratio, from the Genetic Investigation of Anthropometric Traits (GIANT http://portals.broadinstitute.org/collaboration/giant/index.php/Main_Page) [12, 13]. They conducted meta-analyses of GWAS of waist-to-hip ratio adjusted for body mass index in 142762 individuals of European ancestry from 57 GWAS analyses and separately in an additional 67326 European ancestry individuals from 44 cohorts [12]. The meta-analysis of BMI contained 322154 individuals of European descent from 125 studies [13]. The GWAS summary data of waist-to-hip ratio and BMI included 210088 and 322154 European ancestry individuals, respectively.

We obtained GWAS summary data of serum lipids, including HDL-C LDL-C and triglycerides, from The Global Lipids Genetics Consortium (GLGC http://lipidgenetics.org/). A total of 188578 individuals were included in this GWAS [14]. Most of the individuals (169900) are of European ancestry, and as few as 18678 individuals are of other ancestries [14].

All of the GWAS summary statistics adopted in this study are publicly available and freely downloadable. Ethical approval had already been obtained by the original GWAS analyses.

### Mendelian randomization

As described previously, we extracted GWAS summary statistics of traits of interest and aminotransferases from different GWAS analyses. To test the causal relationship between aminotransferase and metabolic syndrome, two-sample MR and bidirectional MR analyses were conducted between aminotransferase and other traits using these GWAS summary statistics. Since the strong horizonal pleiotropy of SNPs related to serum lipids was difficult to eliminate, we conducted a multivariable MR analysis using HDL-C, LDL-C and triglycerides as risk factors.

Mendelian randomization is based on three main assumptions of instrumental variable (IV): (1) the IV is associated with the risk factor, (2) the IV is not associated with confounders, and (3) the IV influences the outcome only by the risk factor [15]. IV in multivariable MR is more or less identical to the one in univariable MR except that IV is associated with a set of risk factors rather than one risk factor [16].

As described in assumption one, genome-wide significant (p<5×10^−8^) SNPs were selected as IVs for MR analyses. The variance in exposures explained by IV was calculated. Because some IVs were missing in the outcome statistics, we selected SNPs in high linkage disequilibrium (r^2^>0.8) as proxies. To examine the statistical power of IV, we calculated F statistics for each IV. IVs with an F statistic less than 10 are usually defined as weak IV and were excluded from this study. To ensure Mendelian inheritance, all SNPs were scrutinized for linkage disequilibrium (LD) (r^2^<0.01). Furthermore, palindromic SNPs and infrequent variants (minor allele frequency<0.01) were removed. To ensure assumptions two and three, SNPs with horizonal pleiotropy need to be removed. Consequently, MR-PRESSO tests were performed to identify and remove SNPs with horizonal pleiotropy. MR-PRESSO tests include the following three tests: global test, distortion test and outlier test. The outliers in MR-PRESSO tests were excluded. All three tests have high power to detect horizonal pleiotropy except for the perfect overlapped samples [17].

### Statistical analysis and visualization

Inverse variance weighted regression model, Egger’s regression (MR Egger) and weighted median were carried out in this study. The result of the inverse variance weighted regression model (IVW) was adopted as the main result in both univariable and multivariable MR analyses. If heterogeneity could be detected, the multiplicative effects model of IVW was adopted. Along with MR-PRESSO tests, the intercept of Egger’s regression was used for testing pleiotropy. The weighted median method will be adopted if most of the IVs are invalid IVs [18]. To test the heterogeneity, the Cochran Q test was carried out. The leave-one-out sensitivity analysis was performed to test the robustness of this study. We tested the direction of causality using MR steiger [19]. The statistical power of this study was calculated using mRnd (https://cnsgenomics.shinyapps.io/mRnd/) [20]. A false discovery rate was adopted to adjust the multiple testing. The effect size of the binary outcome was measured by the odds ratio (OR). Beta (logOR) was adopted for continuous outcome.

All analyses and data visualization were conducted using R version 4.1.1 (https://www.r-project.org/). The R packages ‘TwoSampleMR’, ‘MRPRESSO’ and ‘ggplot2’ were employed in this study.

## Results

As mentioned earlier, our aim was to investigate the causal relationship between aminotransferase and traits related to metabolic syndrome. The evidence suggests that there are bidirectional causal relationships between elevated aminotransferase levels and traits related to metabolic syndrome.

Using the method described above, we filtered IVs and calculated r^2^ (variance explained by IV) for each case. None of the SNPs were weak instrumental variables with F statistics less than 10. In most cases, r^2^ ranged from 0.005 to 0.229. Except for some traits (NAFLD, fasting insulin, fasting glucose and HbA1c) that lacked the effect allele frequency to calculate the power, the statistical powers for all significant results were 100%. Details of the identified SNPs, r^2^ and statistical power are provided in S1-S4 Tables of S1 File. Since the direction of aminotransferases on NAFLD did not pass the Steiger filtering analysis, they were removed from the results. Because heterogeneity was detected in this study, IVW (multiplicative random effects model) was adopted as the main method. Most pleiotropy was eliminated by three tests of MR-PRESSO. Because the strong horizonal pleiotropy between the serum lipids cannot be easily eliminated, the effect estimates of multivariable MR were preferred.

### Effect estimates of aminotransferase on outcomes

**Fig 1:**
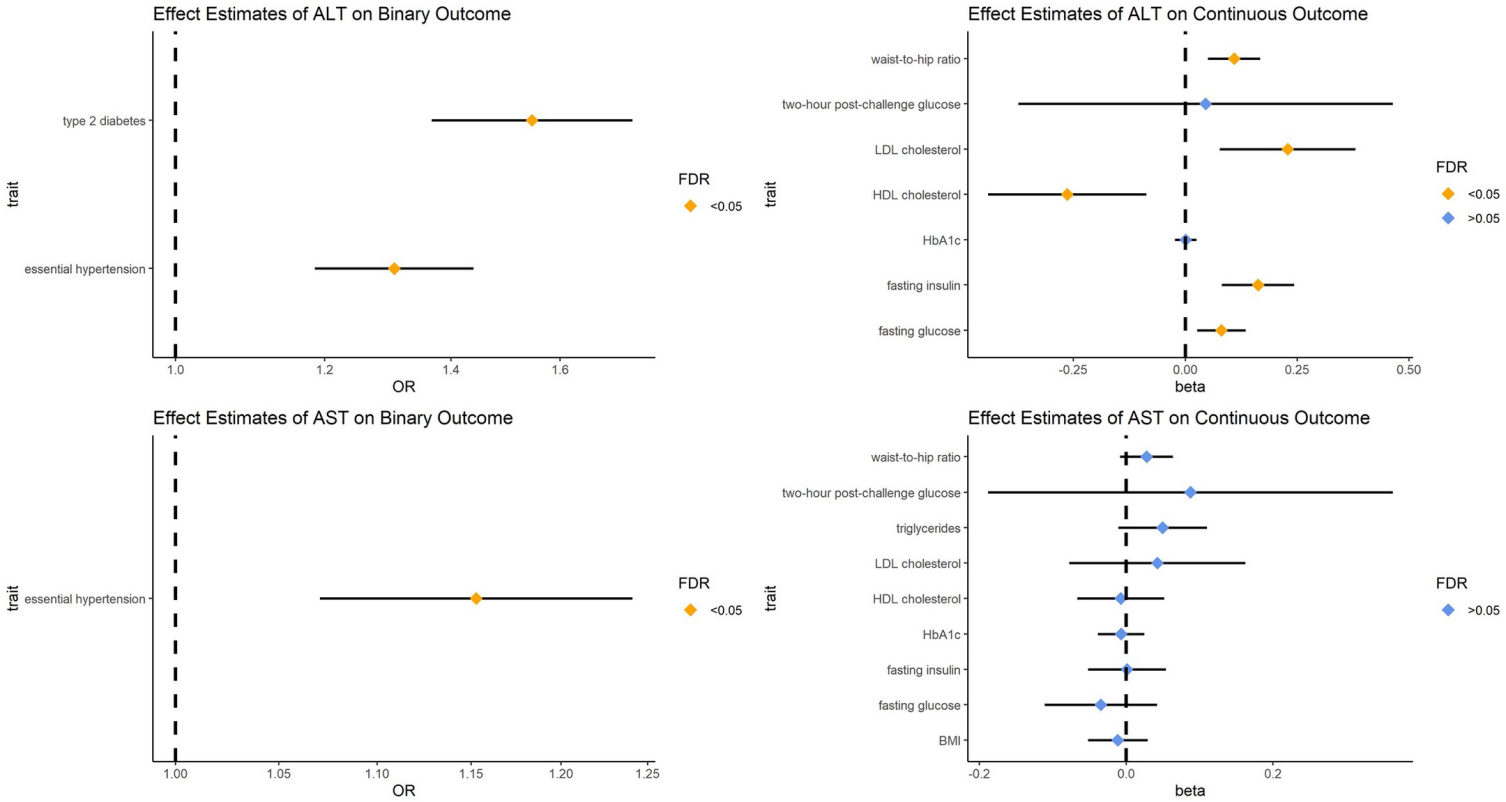
Forest plots for the causal effect of aminotransferases on different traits. OR: odds ratio; Beta = logOR. The results with pleiotropy and that could not pass the Steiger filtering were excluded.

As illustrated in Fig 1, the causal estimates of ALT on binary outcomes were statistically significant with FDR<0.05. Partially in line with a previous study [21], the odds of essential hypertension (OR = 1.306 [1.186,1.439], P = 6.26×10^−8^) and type 2 diabetes (OR = 1.546 [1.367,1.748], P = 3.43×10^−12^) increased per 1-SD increase in ALT levels. For continuous outcomes, the elevation of ALT was a risk factor for most traits. In traits of hyperglycemia, the levels of fasting glucose (beta = 0.081 [0.026,0.135], P = 3.53×10^−3^) and fasting insulin (beta = 0.163 [0.082,0.244], P = 8.14×10^−5^) increased per 1-SD increase in ALT. Among the serum lipids, the causal effects of ALT on HDL-C and LDL-C were totally opposite. The level of LDL-C (beta = 0.229 [0.077,0.380], P = 3.08×10^−3^) was positively associated with ALT, while HDL-C (beta = -0.264 [-0.441, -0.087], P = 3.46×10^−3^) was negatively associated with ALT. Among the two traits related to obesity, the effect of ALT on waist-to-hip ratio (beta = 0.110 [0.051–0.168], P = 2.60×10^−4^) was statistically significant, indicating that the elevation of ALT could give rise to abdominal obesity. The evidence suggested that ALT could be a risk factor for metabolic syndrome.

As shown in Fig 1, AST cannot affect continuous outcomes significantly. The increase in AST levels only elevated the odds of essential hypertension (OR = 1.153 [1.071,1.241], P = 1.63×10^−4^). Although the effect of AST on metabolic syndrome was not significant, a positive effect of AST on blood pressure was found in this study.

### Effect estimates of exposures on aminotransferase

**Fig 2:**
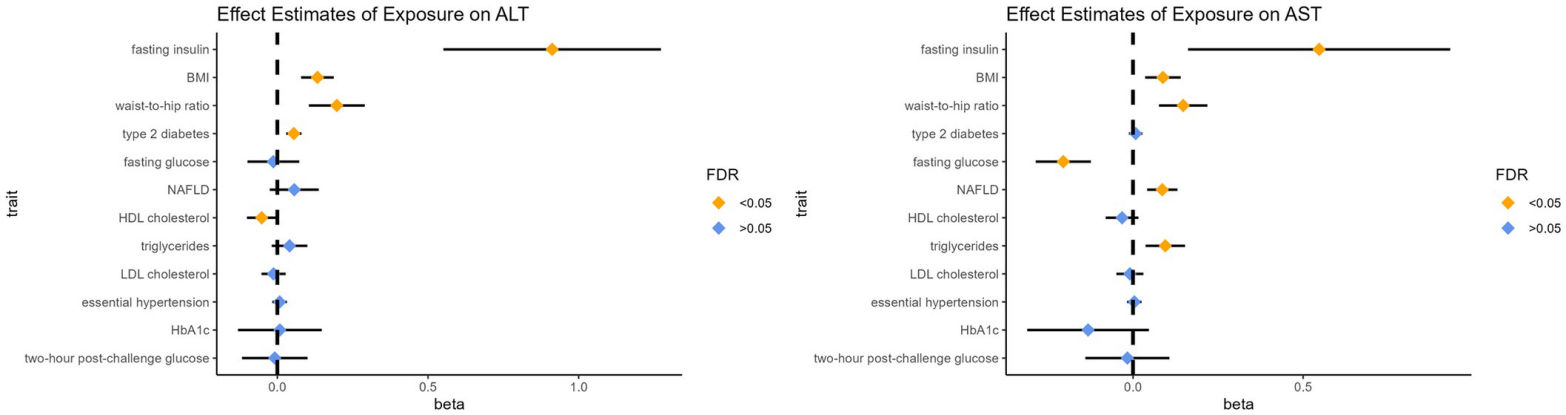
Forest plot for the causal effect of exposure on aminotransferases. The results of multivariable MR were presented for lipids. Others are the results of univariable MR.OR: odds ratio; Beta = logOR. The results with pleiotropy and that could not pass the Steiger filtering were excluded.

As illustrated in Fig 2, waist-to-hip ratio (beta = 0.198, P = 3.02×10^−5^; beta = 0.147, P = 5.01×10^−5^), BMI (beta = 0.133, P = 1.47×10^−6^; beta = 0.088, P = 9.59×10^−4^) and fasting insulin (beta = 0.912, P = 7.31×10^−7^; beta = 0.547, P = 5.40×10^−3^) showed consistent positive effects on the elevation of ALT and AST. Regarding the other traits, type 2 diabetes (beta = 0.055 [0.029, 0.080], P = 2.31×10^−5^) had a positive effect on the elevation of ALT. NAFLD (beta = 0.086 [0.041, 0.131], P = 1.56×10^−4^) was a risk factor for circulating AST, while hypertension did not affect either ALT or AST. Fasting glucose showed a negative effect on AST (beta = -0.205 [-0.286, -0.124], P = 7.16×10^−7^). Among serum lipids, the results of multivariable MR showed that HDL-C (beta = -0.052 [-0.101, -0.003], P = 3.83×10^−2^) could reduce the level of ALT, while triglycerides (beta = 0.095 [0.037, 0.153], P = 1.35×10^−3^) could elevate the level of AST. In other words, atherogenic dyslipidemia could be a risk factor for the elevation of aminotransferases.

The results of IVW (multiplicative random effects model) are visualized in Figs 1 and 2. Details of the univariable MR results are provided in S5-S8 Tables of S1 File. The results of multivariable MR are provided in S9 and S10 Tables of S1 File.

## Discussion

As mentioned in the introduction, our aim is to describe and examine the relationship between aminotransferases and metabolic syndrome. As anticipated, ALT is a risk factor for most outcomes, while the effects of AST on outcomes are not noticeable except for essential hypertension. It is evident from the results that both ALT and AST could be affected by insulin resistance, atherogenic dyslipidemia and obesity.

Among the binary traits, the positive effects of aminotransferases on essential hypertension were confirmed in this study. Because we selected hypertension rather than blood pressure as the outcome, it is apparent that the elevation of ALT could not only contribute to the elevation of blood pressure within the normal range but also result in hypertension. The specific mechanism remains uncertain. Nevertheless, several observational studies have lent support to our results. A case–control study based on a Chinese ancestry population strongly suggested that ALT is an indicator of hypertension [22]. Similar results were also found in a cross-sectional study in Bangladesh adults [23]. However, an opposite result was proposed by Liu et al. [24]. A negative association between blood pressure and aminotransferases was found in young soldiers [24]. Because our analyses were based on a normal European ancestry population while the research of Liu was based on young military samples of Asian ancestry, the effects of aminotransferases on blood pressure may vary between different ages. Furthermore, our study demonstrated that essential hypertension could affect the level of aminotransferases. This result expands the conventional effect of hypertension. Partially consistent with previous studies, we found that NAFLD could affect the level of AST rather than ALT. It is well documented that the level of ALT in NAFLD patients is normal more often than not. Ma et al. argued that at least 25% of NAFLD patients possess normal ALT in the clinical manifestation [25]. It can be inferred that the role of ALT in metabolic syndrome cannot be attributed to NAFLD. As reported previously, ALT could affect most outcomes, while AST could not. It can be concluded that NAFLD is a manifestation of metabolic syndrome, not a cause. For type 2 diabetes, our result is partially in line with the result of a previous MR study conducted by Silva et al. [21]. They believed that both circulating levels of ALT and AST were associated with type 2 diabetes [21]. As illustrated in Figs 1 and 2, bidirectional causality between type 2 diabetes and ALT was demonstrated in this study, while we disproved the causal relationship between AST and type 2 diabetes. Although both hypertension and type 2 diabetes are manifestations of metabolic syndrome, AST affects them differently. The differences between ALT and AST could be attributed to the different distributions in the human body. AST is found in the liver and several types of organs, while ALT exists mainly in the liver [26]. It can be assumed that the interaction between aminotransferases and type 2 diabetes (or metabolic syndrome) is associated with liver function.

In continuous traits, all three groups of traits, including hyperglycemia, serum lipids and obesity, can be affected by ALT, while there seems to be no effect of AST on them. Although both aminotransferases are biomarkers of liver injury, they play different roles in the development of metabolic syndrome.

Among the traits related to hyperglycemia, ALT is a risk factor for fasting glucose and fasting insulin; on the other hand, HbA1c and two-hour post challenge glucose cannot be affected by ALT. It can be inferred that ALT contributes to the early stage of the development of metabolic syndrome, especially insulin resistance. Although ALT is a risk factor for fasting glucose, it may not result in a persistent elevation of blood glucose because of no effect on HbA1c. The effects of hyperglycemia-related traits on different aminotransferases are largely the same except for fasting glucose. This result is partially in line with the Australian population-based study, which suggested that ALT was associated with metabolic syndrome independent of insulin resistance [6]. Our study proves that insulin resistance might affect the levels of ALT and AST to some extent, while only the elevated level of ALT could contribute to the development of insulin resistance as well as metabolic syndrome. We also corroborate that fasting insulin has an indirect effect on essential hypertension mediated by AST. It can be speculated that ALT has appreciable interactions with insulin resistance, while the elevation of AST is one of the manifestations for the downstream effect of insulin resistance. This evidence reinforced that insulin resistance is a main cause of metabolic syndrome, as reported in a previous study [27]. Interestingly, compared to the large effect size of fasting insulin on aminotransferases, fasting glucose showed a negative effect on the level of AST. This could be due to the different effects of blood glucose on different organs. The effect of hyperglycemia on AST is still confusing. Further studies are needed.

For serum lipids, the elevated level of ALT could be a cause of dyslipidemia, including the decrease in HDL-C and the increase in LDL-C. Only triglycerides cannot be affected by ALT. This could be due to the pleiotropy (Egger’s intercept = 0.009, P = 0.02) of IVs selected for ALT to estimate the causal effect of ALT on triglycerides, which cannot be eliminated by removing the outliers. As illustrated in Fig 2, after adjustments for other lipids, the effect of HDL-C on ALT and the effect of triglycerides on AST remained significant. These results verify the associations between serum lipids and ALT described in a previous study [28] and further prove the effects of atherogenic dyslipidemia on AST.

Finally, among the traits related to obesity, only the waist-to-hip ratio (adjusted for BMI) could be elevated by ALT, while BMI was not. It is apparent that compared to normal obesity, abdominal obesity is more likely to be affected by ALT. Abdominal obesity is one of the basic features of metabolic syndrome. As detailed in Fig 2, the positive effects of obesity (BMI) and abdominal obesity (waist-to-hip ratio) on aminotransferases were demonstrated by this study. These findings are partially consistent with previous studies [7, 28]. However, they only emphasized the relationship between ALT and BMI but overlooked the effect of waist-to-hip ratio demonstrated in this study. Moreover, our study confirms the mediation effect of AST between obesity (BMI, waist-to-hip ratio) and essential hypertension. This study provides novel insight into the mechanism of metabolic syndrome.

Our study introduced the MR approach to investigate the causal relationships between aminotransferases and metabolic syndrome. We not only treated aminotransferases as biomarkers but also emphasized the effect of aminotransferases. Several causal relationships have been demonstrated in this study. One of the most compelling results is the positive effects of aminotransferases (both ALT and AST) on essential hypertension. The reciprocal causality between ALT and type 2 diabetes implies that ALT could be a biomarker of type 2 diabetes. The causal relationships between aminotransferases and the other three main traits (hyperglycemia, dyslipidemia, obesity, and especially abdominal obesity) were corroborated in this study. It is evident from the result that metabolic syndrome could affect the level of aminotransferases. The opposite effect of fasting insulin and fasting glucose was described in this study. Specific mechanisms should be explored in future work.

However, our study has some limitations that should be considered when interpreting the results. First, the MR analysis assumes that the IVs are independent of the confounders and only affect the outcome through the exposure. However, this assumption may be violated by the presence of population stratification, horizontal pleiotropy, or linkage disequilibrium. We tried to minimize these biases by using multiple methods to select the IVs, such as clumping, pruning, and MR-Egger regression. Second, the MR analysis assumes that the exposure is independent of the outcome and does not affect the IVs. However, this assumption may be violated by the presence of reverse causality, feedback loops, or time-varying effects. We tried to address these issues by using bidirectional MR and two-sample MR with different data sources. Third, the MR analysis may have limited generalizability and applicability due to the use of essential hypertension instead of blood pressure as the outcome, the lack of minor allele frequency data, and the heterogeneity of the study populations. We suggest further studies to validate our findings in different ethnic groups, using continuous blood pressure measurements, and accounting for the minor allele frequency variations.

In conclusion, most of the causal effects of ALT on metabolic syndrome-related traits are statistically significant. There could be a reciprocal causation between metabolic syndrome and ALT. Metabolic syndrome could elevate the level of AST. Both circulating ALT and AST enhance the incidence of essential hypertension, and the former could also increase the incidence of type 2 diabetes. The elevation of aminotransferases in people without liver injury could be an early signal of metabolic syndrome, especially for essential hypertension.

## Supporting information

S1 File Supplementary tables identified. (XLSX)

## Abbreviations

ALT: Alanine aminotransferase
AST: Aspartate aminotransferase
GIANT: Genetic Investigation of Anthropometric Traits
GLGC: Global Lipids Genetics Consortium
GWAS: genome-wide association study
HDL-C: HDL cholesterol
LDL-C: LDL cholesterol
MAGIC: Meta-Analyses of Glucose and Insulin-related Traits Consortium
MR: Mendelian randomization
MR–Egger: Mendelian randomization-Egger
MR-PRESSO: Mendelian Randomization Pleiotropy RESidual Sum and Outlier
